# Microimaging of a novel intracochlear drug delivery device in combination with cochlear implants in the human inner ear

**DOI:** 10.1007/s13346-021-00914-9

**Published:** 2021-02-04

**Authors:** Eric Lehner, Matthias Menzel, Daniel Gündel, Stefan K. Plontke, Karsten Mäder, Jessica Klehm, Heike Kielstein, Arne Liebau

**Affiliations:** 1 Institute of Pharmacy, Martin Luther University Halle-Wittenberg, Halle (Saale), Germany; 2 Fraunhofer Institute for Microstructure of Materials and Systems (IMWS), Halle (Saale), Germany; 3 Department of Nuclear Medicine, Martin Luther University Halle-Wittenberg, Halle (Saale), Germany; 4 Department of Otorhinolaryngology-Head and Neck Surgery, Martin Luther University Halle-Wittenberg, Halle (Saale), Germany; 5 Institute of Anatomy and Cell Biology, Martin Luther University Halle-Wittenberg, Halle (Saale), Germany

**Keywords:** Biodegradable polymer, PLGA, Temporal bone, Cochlear implant, μCT, Inner ear drug delivery

## Abstract

The effective delivery of drugs to the inner ear is still an unmet medical need. Local controlled drug delivery to this sensory organ is challenging due to its location in the petrous bone, small volume, tight barriers, and high vulnerability. Local intracochlear delivery of drugs would overcome the limitations of intratympanic (extracochlear) and systemic drug application. The requirements for such a delivery system include small size, appropriate flexibility, and biodegradability. We have developed biodegradable PLGA-based implants for controlled intracochlear drug release that can also be used in combination with cochlear implants (CIs), which are implantable neurosensory prosthesis for hearing rehabilitation. The drug carrier system was tested for implantation in the human inner ear in 11 human temporal bones. In five of the temporal bones, CI arrays from different manufacturers were implanted before insertion of the biodegradable PLGA implants. The drug carrier system and CI arrays were implanted into the scala tympani through the round window. Implanted temporal bones were evaluated by ultra-high-resolution computed tomography (µ-CT) to illustrate the position of implanted electrode carriers and the drug carrier system. The µ-CT measurements revealed the feasibility of implanting the PLGA implants into the scala tympani of the human inner ear and co-administration of the biodegradable PLGA implant with a CI array.

**Graphical abstract:**

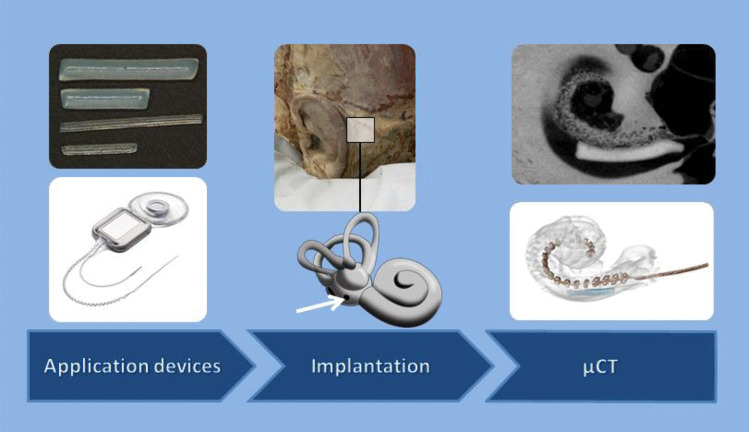

## Introduction

Delivering drugs to the cochlea in effective doses is still a major unsolved problem in the treatment of inner ear diseases, such as idiopathic sudden sensorineural hearing loss, noise-induced hearing loss, and Ménière's disease [1, 2]. Insufficient drug concentrations after intravenous injection or oral intake in particular are the result of first pass metabolism, barrier effects of the blood–labyrinth barrier, and restrictions on systemic concentrations either because of the need to avoid toxic side effects or cost-related measures (low amount of drug available) [2, 3]. Local drug delivery to the inner ear could principally overcome these issues of systemic administration. In the most widely used form of intratympanic, extracochlear drug delivery, a drug solution is injected trough the tympanic membrane into the middle ear. Afterwards, the drug reaches the inner ear by diffusing through the round window membrane and the oval window [4]. For effective drug transfer to the inner ear, close contact of the drug solution with the round window membrane and/or stapes footplate and long drug persistence are important. Additional mucosal folds (“false round window membranes”), rapid clearance of the drug from the middle ear, and the diffusion barriers of the round window membrane and oval window are the primary obstacles in achieving suitable drug concentrations [1, 5, 6]. Detailed discussions of the main drug delivery systems were published recently [2, 3, 7–10].

Intracochlear drug delivery, in which the drug is directly released into the inner ear fluid, would overcome the limitations of both systemic and extracochlear drug delivery. However, this method of application is currently restricted to cochlear implant (CI) surgery when the cochlea is opened to insert the electrode carrier. A primary research focus in intracochlear drug delivery is the development of drug-releasing electrode carriers [11–16]. However, the fixed combination of both systems leads to limitations in the drug delivery system, as the matrix material is determined by its main function of being the electrode carrier. For an independent intracochlear drug delivery device, the choice of matrix material would be more flexible, allowing better control of release kinetics and expanding the range of usable drugs. The alternative approach with an independent drug delivery system offers an opportunity for personalized medicine because different drugs and different amounts could be adjusted independently from the chosen CI device. Furthermore, an intracochlear drug carrier system could be used independently from CIs for the application of substances used in treatments that cannot be applied systemically or via extracochlear application due to one of the above restrictions. To avoid additional surgical trauma during removal of the drug delivery system, the device should dissolve after termination of the drug treatment and, therefore, be completely biodegradable.

We have developed a biodegradable drug carrier system for drug release in the inner ear based on a mixture of poly(D,L-lactic-co-glycolic acid) (PLGA) and polyethylene glycol (PEG). The manufacture of the drug carrier system and release kinetics for dexamethasone were described previously [17]. The measurement of in vitro drug release and additional mathematical simulations of the in vivo release kinetics in the human inner ear suggest that the drug carrier system can achieve controlled and sustained drug levels without an initial lag phase [17]. For further development of this system in the direction of clinical use, general proof and the limits of possible administration to the human cochlea are needed. Therefore, in the present study, four different sizes (variation in diameter and length) of the drug carrier system were tested in human cadaver temporal bones. BaSO_4_-loaded implants containing the drug carrier system were implanted into the scala tympani through the round window. In five temporal bones, CI electrode arrays from different manufacturers were implanted before insertion of the drug delivery system in order to test whether it is possible to use the drug delivery device for simultaneous local drug therapy. After implantation, the temporal bones were evaluated by ultra-high-resolution computed tomography (µCT) to illustrate the position of the CI array and drug carrier.

## Materials and methods

### Materials

PLGA (Expansorb® DLG 50-2A) was provided by Merck KGaA (Darmstadt, Germany). PEG (1500 g mol^−1^) was purchased from Alfa Aesar (Haverhill, USA). Barium sulfate nanoparticles (D90 = 0.35 µm; Blanc Fixe® Solvay, Massa, Italia) were used as radiopaque markers. Dulbecco’s phosphate-buffered saline (8 g/L NaCl, 0.2 g/L KCl, 1.15 g/L Na_2_HPO_4_, 0.2 g/L KH_2_PO_4_) adjusted to pH 7.4 was used for stability measurements. Sodium azide 0.02% was added to the phosphate buffer to avoid microbial growth. CI electrode carriers (Table 1) were provided by the respective manufacturers (MED-EL, Cochlear, Advanced Bionics). Formaldehyde-preserved human temporal bones from body donors were provided by the Institute of Anatomy and Cell Biology, Martin Luther University Halle-Wittenberg, Halle (Saale), Germany.

**Table 1:** Implanted temporal bone characteristics

Temporal bone	PLGA implant dimension	Cochlear implant array
#1	0.3 × 3 mm	None
#2	0.3 × 5 mm	None
#3	0.6 × 3 mm	None
#4	0.6 × 3 mm	None
#5	0.6 × 5 mm	None
#6	0.6 × 5 mm	None
#7	0.3 × 3 mm	Cochlear Contour Advance
#8	0.3 × 3 mm	Advanced Bionics HiFocus Mid-Scala
#9	0.3 × 3 mm	MED-EL FLEXSOFT
#10	0.3 × 3 mm	Cochlear Slim Modiolar
#11	0.6 × 5 mm	Cochlear Slim Modiolar

### Preparation of barium sulfate-loaded extrudates

PLGA, PEG, and barium sulfate were pulverized in a Cryomill (Retsch GmbH, Haan, Germany) for 90 s in 4 cycles at a frequency of 15 Hz to obtain homogeneous material. The grinding jar was continually cooled with liquid nitrogen. The powder was removed when it reached room temperature. The pre-mixed powder was then manually fed into the nitrogen air-cooled barrel of a Three-Tec twin-screw extruder (ZE 5 ECO; Three-Tec GmbH; Seon; Swiss). The set points of the three heating zones, from feed to die, were 50, 50, and 52 °C, respectively, and screw speed was maintained at 60 rpm throughout. A 0.3 mm and 0.6 mm die were used. The extruded material was collected and stored in a fridge at 2–8 °C.

### Macroscopic characterization

The size and morphology of the PLGA implants were studied using an Olympus SZX9 microscope with a UC30 camera (Olympus Optical Co., Hamburg, Germany) and OLYMPUS stream motion software (Olympus Optical Co., Hamburg, Germany). The length of each implant was adjusted to 3.0 mm or 5.0 mm by cutting the extrudates under the microscope with a scalpel. A tolerance of 0.1 mm was accepted.

### Limit of detection in the cochlea

The detectability of the PLGA implant with respect to the surrounding bony tissue in the cochlea was determined by placing PLGA implants measuring 0.3 mm × 3 mm in 1 mL PBS (pH 7.4) at 37 °C. The buffer solution was refreshed daily. Measurements were carried out at several time points (day 0, 3, 7, and 28) using a small animal nanoScan PET/CT (Mediso GmbH, Münster, Germany) with 720 projections and an X-ray energy of 70 kVp. For the reconstruction (voxel size: 25 µm × 25 µm × 25 µm, filter: cosine) of CT images, Nucline Software (Mediso GmbH, Münster, Germany) was used. The reconstructed images were analyzed using Pmod Software (PMOD Technologies LLC, Zürich, Schweiz), and the volume of interest (VOI) of the PLGA implants depicted by an intensity-based threshold algorithm. The calculated Hounsfield Units (HU) were compared with published human temporal bone data [18]. Each experiment was conducted in triplicate.

### Implantation of temporal bones

All surgical procedures were performed in the central operating theaters of our university hospital under the same conditions and using instruments sets as in standard middle and inner ear surgery. The specimens were placed on an operating table, and the operating field was visualized with fully digital microscope (ARRSICOPE, Munich Surgical Immaging GmbH, Munich, Germany). All surgical procedures were done by an experienced otologic and cochlear implant surgeon (SKP). PLGA implants were implanted using a transmeatal approach, i.e., via the external auditory canal. After raising a tympanomeatal flap, the round window niche was identified and an often present bony overhang form the promontory of the cochlea was removed using an otological high-speed drill until the round window membrane was fully visible. This can be considered a standard surgical procedure for trained otological surgeons. The round window membrane was incised using a 0.4 mm 90° hook. The drug delivery system was inserted through the round window membrane with alligator forceps, followed by gently pushing it into scala tympani with an otological needle. In temporal bones with implanted CI electrode arrays, the arrays were inserted through the round window first. For electrode arrays with a larger diameter (#7), the round window was slightly enlarged anteriorly and inferiorly as in standard CI surgery. After insertion of the CI array, the drug delivery device was placed next to the electrode carrier in the basal part of the basal turn of the cochlea in the scala tympani. The dimensions of the inserted PLGA implants and CI arrays for each implanted temporal bone are provided in Table 1.

### X-ray computed tomography

For visualization and evaluation of PLGA implants in combination with CI arrays regarding the location and integrity after implantation, µCT-microscopy was applied (RayScan200E, RayScan Technology GmbH, Meersburg, Germany). To achieve the best possible resolution without dissecting the temporal bone, only the area located around the cochlea was scanned (ROI-Scan). For each specimen, a minimum of 2970 2D-projections at a full 360° rotation were recorded to obtain a voxel resolution after reconstruction at least of 26 µm. The X-ray tube voltage and current were adapted between 180 and 200 kV/80–100 µA to individual specimens to maximize the material contrast while minimizing typical methodic artifacts. Segmentation of the morphological features was performed manually to obtain the three-dimensional structure of the cochlea and implants using VG-Studio max 3.3 (Volume Graphics, Heidelberg, Germany).

## Results and discussion

### Macroscopic characterization

**Fig. 1:**
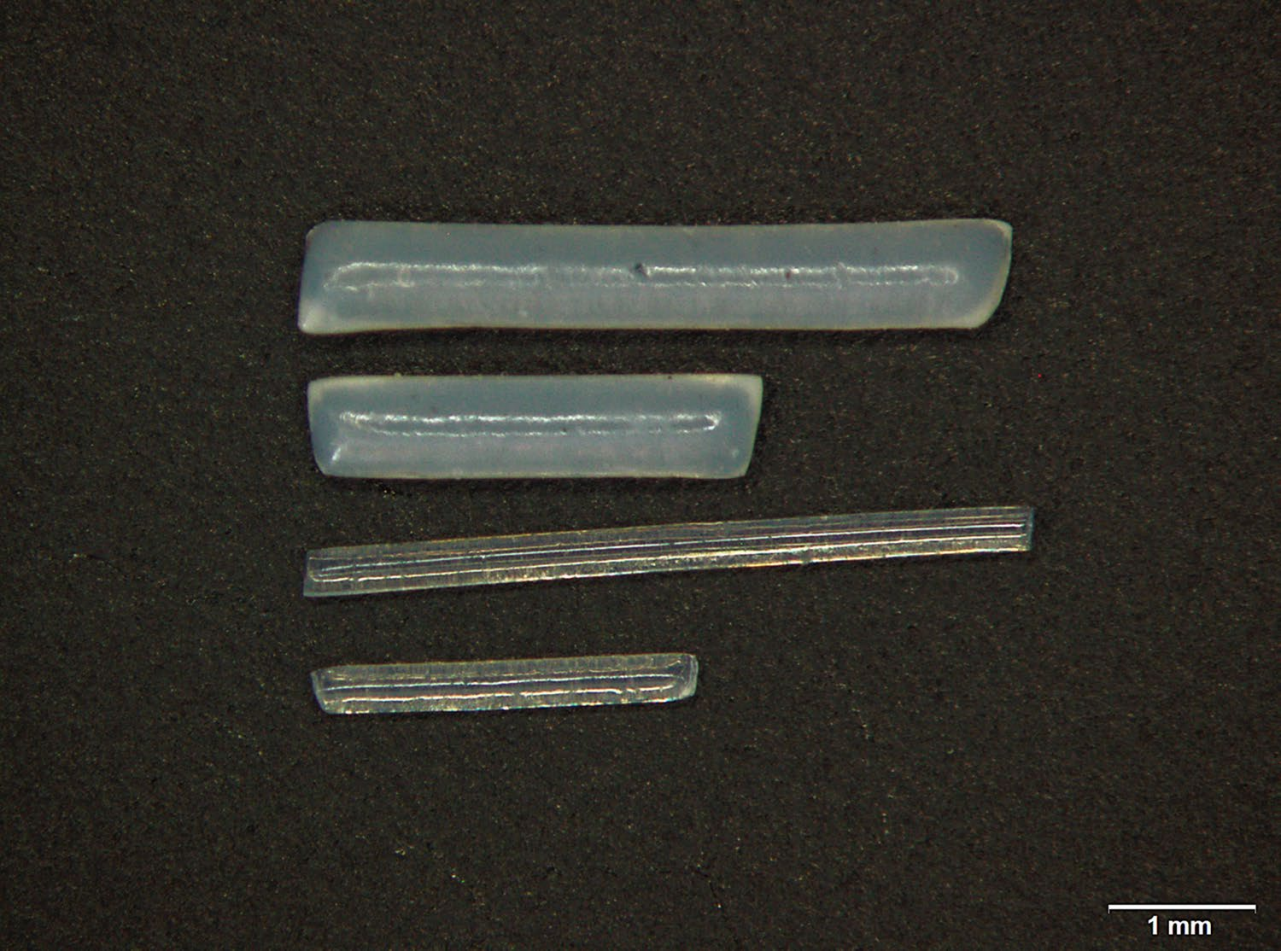
Morphology of BaSO_4_-loaded PLGA implants. Approximate dimensions from top to bottom: 5 × 0.6 mm, 3 × 0.6 mm, 5 × 0.3 mm, and 3 × 0.3 mm

Four different sizes of PLGA implants were selected for cochlear application. The dimensions of the applicable implants are shown in Fig. 1. The 0.3 mm × 3 mm PLGA implants have already been applied successfully in the cochlea of a guinea pig [17]. We wanted to test whether the scala tympani would be injured by increasing the diameter of the implants from 300 to 600 μm. In addition, the length was increased from 3 to 5 mm to determine if the implants were already in the loop of the cochlea as the length of the implants increased. All PLGA implants were rod-shaped and exhibited an opalescence, which is proof of the small size of the BaSO_4_ nanoparticles.

### Limit of detection

**Fig. 2:**
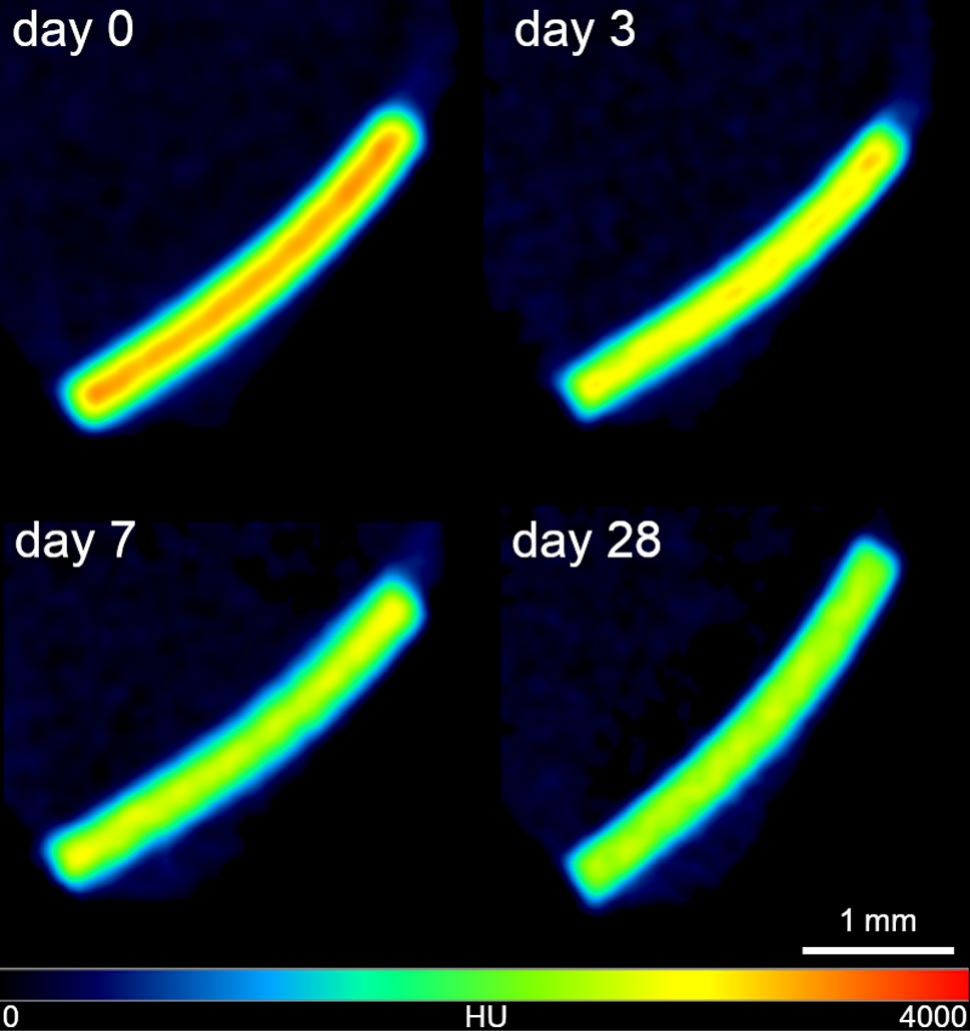
CT Image in hot/cold view of a 0.3 × 3 mm PLGA implant on day 0 and after incubation in PBS on days 3, 7, and 28

**Fig. 3:**
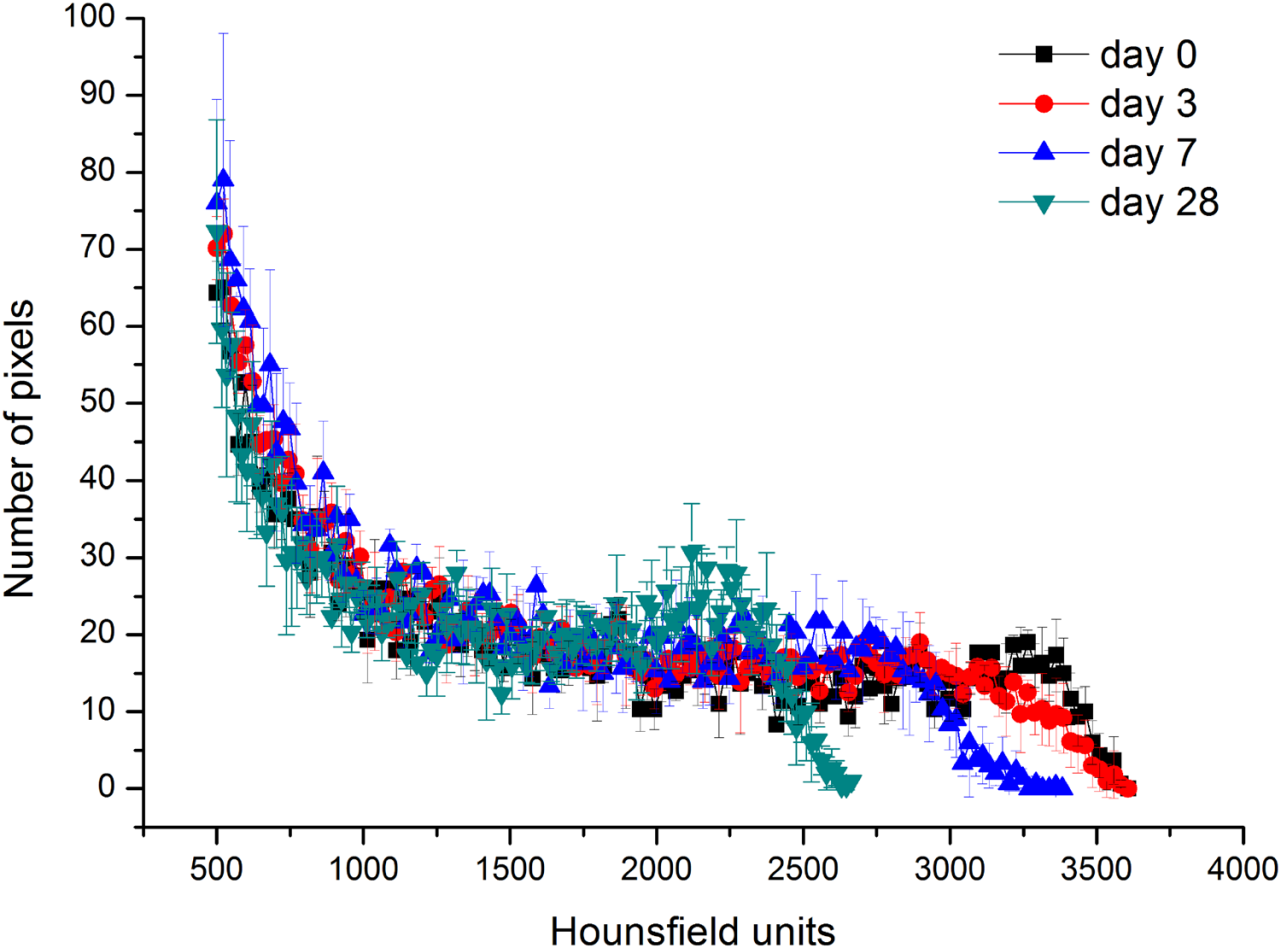
Histogram of the VOIs of the 0.3 × 3 mm PLGA implants in PBS over 28 days

The detectability of the PLGA implants was checked in PBS. We measured the density of the implants in hounsfield units (HU) over 28 days to determine the optimal time for CT measurement in humans. The implants were detected over 28 days, but with a slight decrease in intensity (Fig. 2). The histograms of the VOIs of the implants were compared with published data from the human temporal bone. The maximum was found in the apex cochleae (2703 HU) [18]. Compared with the apex cochleae, the implants were significantly denser on days 0 and 3, with a maximum between 3000 and 3500 HU (Fig. 3). On day 28, the contrast was already so low that all VOIs were similar to the temporal bone. In conclusion, the optimal time for CT after implantation is in the first 3 days. Thereafter, it may be difficult to distinguish the PLGA implants from the surrounding tissue.

### µCT images

**Fig. 4:**
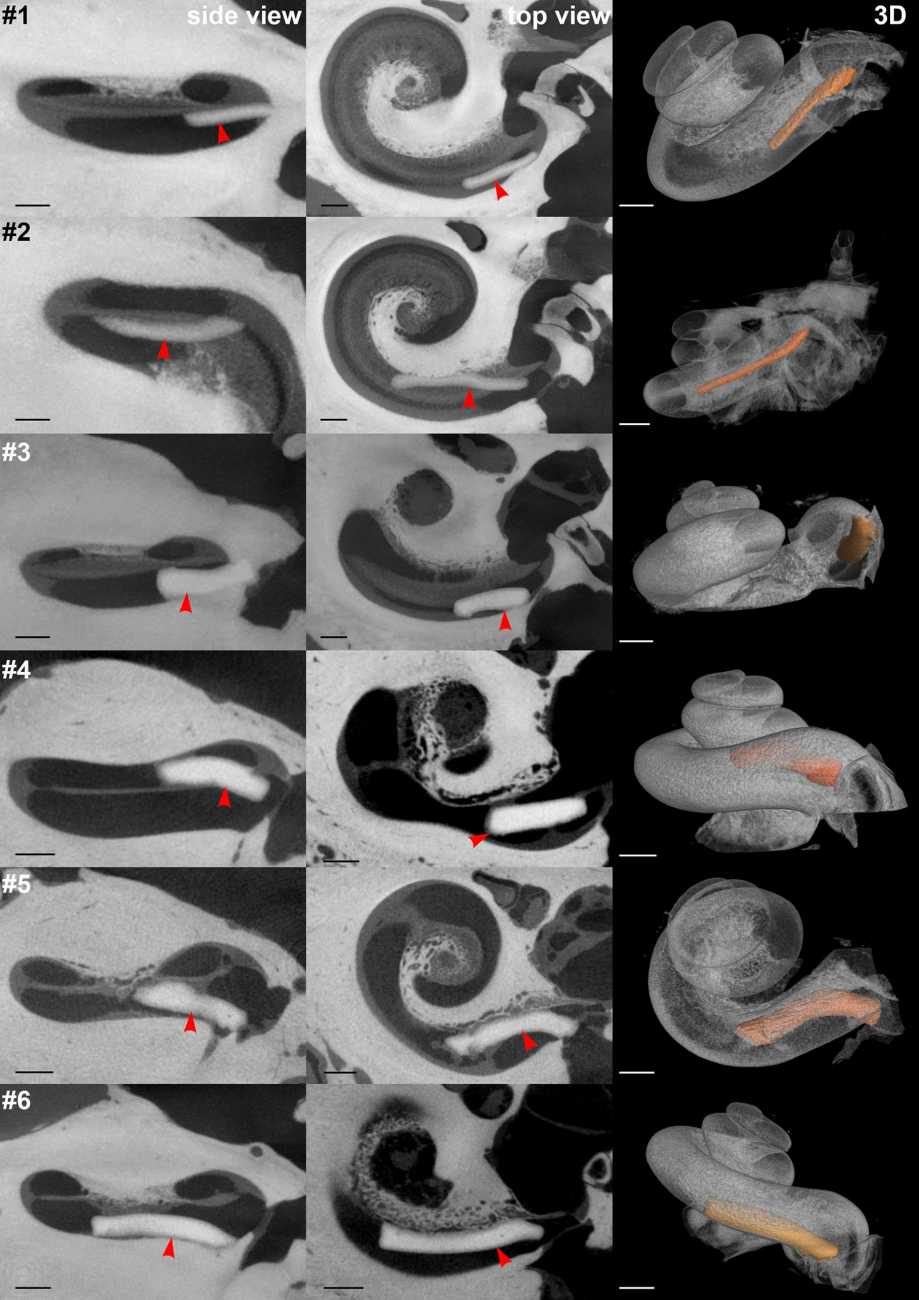
Top view (left), side view (middle), and reconstructed 3D (right) µCT images of human temporal bones implanted with BaSO_4_-loaded PLGA implants. Red arrows indicate the positions of the PLGA implants, which are colored in orange in the 3D reconstruction pictures. Black and white scale bars indicate 1 mm

In six temporal bones (#1–#6), only PLGA implants of different dimensions were implanted into the scala tympani. Figure 4 shows the µCT images of the temporal bones and a 3D reconstruction; there was enough space in the scala tympani for placement of any of the tested dimensions, even implants with a diameter of 0.6 mm. However, in temporal bones #4 and #5, the thicker PLGA implants penetrated the basilar membrane. A scala change can clearly be seen in the respective 3D reconstructions. In contrast, the length of the PLGA implants per se did not seem to have increased the risk for damage of the basilar membrane, as no general difference in the position was observed for the two thinner PLGA implants (0.3 mm diameter, #1 and #2). The PLGA implants demonstrated variable attachment to the scala walls. In terms of safety, it would be advantageous if the PLGA implants could be placed on the lower part of the lateral wall most distant to the basilar membrane and, thus, the organ of Corti and modiolus with Rosenthal’s canal.

**Fig. 5:**
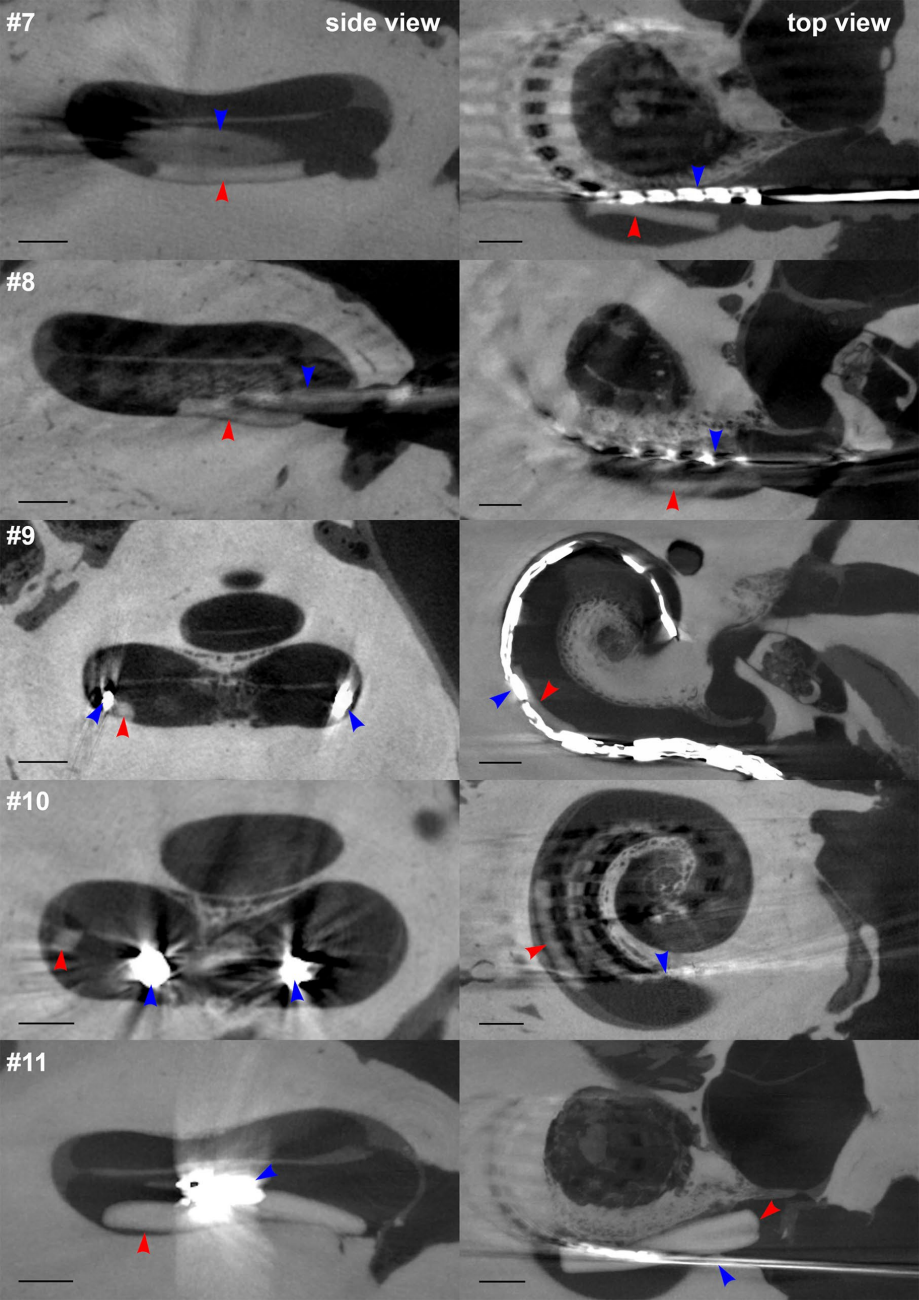
Side view (left) and top view (right) µCT images of human temporal bones implanted with BaSO_4_-loaded PLGA implants and various cochlear implant electrode arrays from different manufacturers. Red arrows indicate the positions of the PLGA implants. Blue arrows indicate the positions of the cochlear implant electrode arrays. Black scale bars indicate 1 mm

**Fig. 6:**
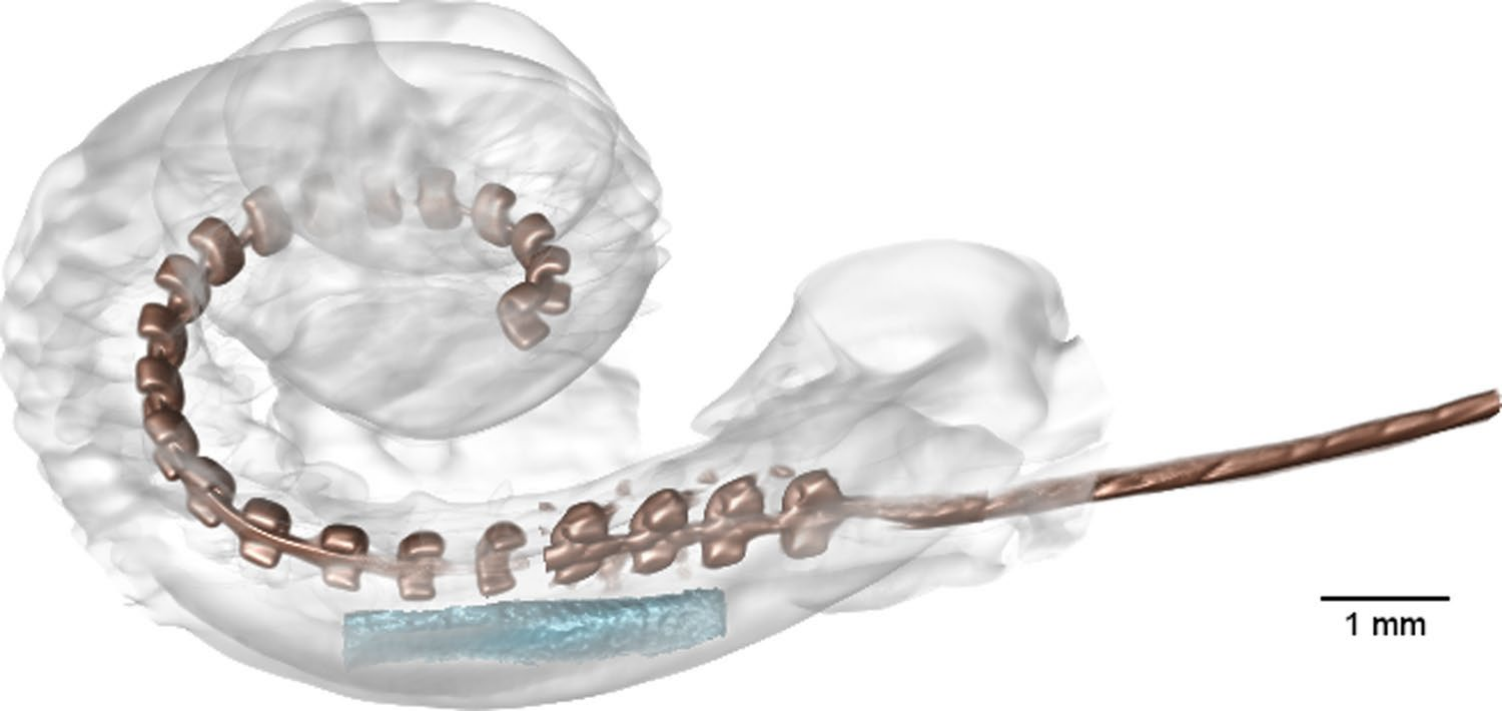
3D reconstruction of the cochlea of temporal bone #7 implanted with a Cochlear Contour Advance electrode (brown) and a BaSO_4_-loaded PLGA implant (0.3 × 3 mm) (light blue)

Figure 5 shows the respective µCT images for the temporal bones with both a PLGA implant and a CI electrode array implanted in the same inner ear (#7–#11). Due to artifacts from metallic components in the electrode arrays, visual detection of the PLGA implants was more difficult. The artifacts could be reduced by slightly lowering the resolution. The images demonstrate enough space in the scala tympani for additional implantation of the PLGA implants despite the already implanted CI arrays. This can also be seen in Fig. 6, which shows a 3D reconstruction of temporal bone #7 implanted with a Cochlear Contour Advance electrode and a PLGA implant 0.3 mm in diameter and 3 mm in length. Due to metallic artifacts, 3D reconstruction was only possible for this temporal bone. In temporal bone #11, the largest PLGA implant tested (0.6 mm × 5 mm) was implanted together with a CI array. The µCT images showed that there was enough space for the two devices, at least in the basal part of the scala tympani. In temporal bones #9 and #10, the PLGA implants were located deeper in the cochlea than in the other temporal bones. This may be an artifact of the PLGA implants attached to the CI electrode array, which may have been pushed further into the cochlea while handling the temporal bone during µCT measurements. Videos of all 3D reconstructions are shown in the Electronic supplementary material.

When a drug solution or drug depot is applied to the round window in extracochlear drug delivery, only a small proportion of the drug diffuses through the round window membrane [1]. Substances that have entered the cochlea are distributed within the inner ear but are simultaneously cleared out or absorbed by the surrounding tissue. These processes lead to a basal to apical concentration gradient depending on the diffusion and tissue penetration parameters of the drug used [1, 5, 6]. Drugs with a small clearance half-life in the inner ear perilymph, such as glucocorticosteroids (22.5 min for dexamethasone [4]), will hardly reach the apical region. Prenzler et al. used an intracochlear catheter made of material with similar soft characteristics as a CI electrode array carrier for delivering drugs closer to the cochlear apex. The catheter was used to apply triamcinolone during CI surgery before insertion of the electrode array [19, 20]. However, the usage of such a catheter carries a risk of mechanical trauma to the inner ear comparable to CI insertion itself. In addition, special care must be taken when using the “cochlear catheter” to avoid harmful perilymph pressure waves during insertion and while injecting the drug solution through the catheter into the inner ear.

If longer lasting intracochlear drug application is intended, a drug depot must be placed, with the incorporated drugs being directly eluted into the cochlear fluids. For a fully implanted controlled release system, the maximum substance concentration will occur along the length of the intracochlear depot depending on the type and load of the depot matrix used. Towards the cochlear apex, a basal to apical concentration gradient will still be present following the same pharmacokinetic principles as discussed above. However, intracochlear drug application has been shown to lead to higher concentrations and more uniform distribution with much smaller gradients along the cochlea [21]. When the electrode array of a CI is used as a drug depot, maximum substance concentration could be expected over the length of the implanted device.

A different approach to intracochlear drug delivery independent from CIs are pumping systems [22–24] or drug reservoirs [21, 25] in which the outlet is fixed in the cochlear bone and drains directly into the perilymph. The advantage of this approach is a large reservoir for drugs without the need for space within the cochlea, which could compromise cochlear function through, for example, changes in micro-mechanical properties. However, high technical effort is needed with pumping systems to prevent a harmful increase in perilymph pressure to cochlear structures [26, 27]. Drug reservoirs can be attached to the bony wall of the cochlea with a fixed outlet in the cochlear bone. Drug elution is driven by diffusion of the substance that has to be delivered through the depot matrix [21, 25]. Depending on where the outlet is fixed in the cochlear capsule, a concentration gradient will occur starting from this point. The disadvantage with this kind of drug delivery is the permanently present cochleostomy in which a foreign body is fixed to the bone. This increases the risk for infection and/or a foreign body reaction associated with fibrosis and ossification of the cochlea. In addition, these drug depots are not biodegradable and have to be removed in a second surgery.

Pierstorff et al. developed small drug depots (< 300 µm diameter) loaded with fluticasone propionate that can be injected directly into the cochlea without higher space consumption in the scalae [28]. Calculations based on the drug-release profile predicted a possible treatment time of many months after a single application. However, the depots are not biodegradable and empty coats will remain in the cochlea after termination of drug elution. Such drug application may not be optimal for drug application in combination with CI insertion because perilymph leakage through the insertion site of the implant may flush out the micro depots. Drug depots that are larger in size and remain in place may be more suitable.

In a clinical pilot study including two patients with CIs, two pieces (< 3 mm in length) of the Ozurdex® implant were placed next to and along a CI array in the basal part of the basal turn of the scala tympani for treatment of an increase in CI impedance and decreased speech understanding likely due to a local inflammatory reaction [16]. The Ozurdex® implant is a PLGA-based drug delivery system approved for intravitreal injection, providing sustained delivery of dexamethasone to the eye. That study indicated that there is enough space in the scala tympani of the human inner ear to place a drug delivery system next to a CI. Although the Ozurdex® implant is completely biodegradable, its material properties are not optimal because the matrix is too stiff. In addition, the release profile has a lag time over several days before the start of drug release, and during elution the amount of dispensed drug varies greatly over time [29].

The mechanical matrix properties of the PLGA implant in the present study is more flexible with a softer texture, and the release profile provides constant drug levels over time without an initial lag phase [17]. In previous study, the polymer was mostly degraded after 21 days [30]. However, swelling, drug release, and polymer degradation are slowed down in vivo due to the small amount of perilymph.

Micro-CT images of implanted temporal bones showed the feasibility of placing the drug delivery devices into the scala tympani and next to CI arrays. However, in thicker implants (600 µm), scala changes were seen in some of the implanted temporal bones. CT revealed that the PLGA implants, regardless of dimensions, remained intact after insertion. In none of the implants with shorter and thinner dimensions was damage to the cochlear tissue detected by micro-CT imaging. Limitations of the study include the small number of implanted temporal bones conserved in formaldehyde. Fixation leads to solidification of the tissue and may have made the cochlear structures less vulnerable to mechanical trauma. Safety studies in fresh temporal specimens or animals with inner ear dimensions similar to the human cochlea will be necessary, including histological examination. Repeated measurement at several time points after implantation could provide information on the swelling and degradation as long as the implants can still be detected.

## Conclusion

This study showed for the first time the general suitability of co-administration of intracochlear controlled release biodegradable drug delivery systems of various dimensions with different CI electrode arrays. PLGA implants of all tested dimensions could be implanted into the scala tympani of the human inner ear. However, thicker implants had a higher risk of damaging the basilar membrane. Therefore, the thickness of implants should be restricted to 300 µm in future studies. Although no signs of any harm to the cochlear structures were detected on micro CT imaging for the longer implants, length may also need to be restricted to 3 mm. Implants with such dimensions could easily be inserted into ears implanted with CI electrode arrays, which creates an opportunity for flexible simultaneous drug therapy independent from the CI electrode array used. We propose that this kind of drug-device combination therapy will contribute to personalized medicine in hearing rehabilitation.

## Supplementary Information

Below is the link to the electronic supplementary material.

Supplementary file1 (AVI 10849 KB)

Supplementary file2 (AVI 21741 KB)

Supplementary file3 (AVI 8020 KB)

Supplementary file4 (AVI 9452 KB)

Supplementary file5 (AVI 11550 KB)

Supplementary file6 (AVI 8217 KB)

Supplementary file7 (AVI 6399 KB)

## Data Availability

The data that support the findings of this study are available from the corresponding author [Arne Liebau] on request.
